# Intimate partner violence and maternal antenatal care utilization: is there a dose-response relationship? Findings from the Ethiopian National Demographic and Health Survey

**DOI:** 10.1093/inthealth/ihaf003

**Published:** 2025-02-05

**Authors:** Bazie Mekonnen, Abebe Gebremariam, Negussie Deyessa, John N Cranmer

**Affiliations:** College of Health Sciences, Addis Ababa University, Addis Ababa, Ethiopia; Woodruff Health Sciences Center, Emory University, Atlanta, GA, USA; College of Health Sciences, Bahir Dar University, Bahir Dar, Ethiopia; Emory-Ethiopia, Addis Ababa, Ethiopia; College of Health Sciences, Addis Ababa University, Addis Ababa, Ethiopia; College of Health Sciences, Bahir Dar University, Bahir Dar, Ethiopia; Emory-Ethiopia, Addis Ababa, Ethiopia; Center for the Study of Human Health, Emory University, Atlanta, GA, USA

**Keywords:** antenatal care, intimate partner violence, maternal mortality, sub-Saharan Africa

## Abstract

**Background:**

Maternal mortality in sub-Saharan Africa (SSA) is an enduring public health challenge. Adequate utilization of antenatal care (ANC) services is one strategy to mitigate the problem by identifying and managing pregnancy risks early. Yet, in SSA, uptake of ANC remains low. Intimate partner violence (IPV) may be a deterrent to ANC uptake. We measured the dose–response relationship between IPV and adequate ANC utilization (defined as four or more visits [ANC-4]) using data from the Ethiopia Demographic and Health Survey (EDHS) 2016.

**Methods:**

We used complex sample logistic regression to measure the impact of three IPV subscales (emotional, sexual and physical) on ANC-4 while controlling for sociodemographic, obstetric and women empowerment factors.

**Results:**

A total of 2599 (weighted) currently married or in-union women were included. There was a significant dose–response relationship between IPV and ANC utilization. Emotional (adjusted odds ratio [aOR] 0.78 [confidence interval {CI} 0.64 to 0.97]) and sexual (aOR 0.68 [CI 0.50 to 0.92]) violence decreased ANC-4 uptake while controlling for the covariates.

**Conclusions:**

IPV is common, yet often invisible, in Ethiopia. Health workers may begin directly screening pregnant women for IPV in order to increase targeted support of ANC uptake. This is the first known study to confirm IPV has a dose–response relationship with ANC-4 uptake.

## Introduction

Maternal mortality from preventable causes remains persistently high, especially in the countries of sub-Saharan Africa (SSA). Worldwide, 295 000 mothers died in 2017 from pregnancy-related causes,^1^ with 66% occurring in SSA.^1,2^ For each maternal death, 20–30 more women suffer acute or chronic pregnancy-related complications such as obstetric fistula and depression.^2^ Of these deaths, 70% are due to preventable or readily treatable causes such as postpartum haemorrhage (PPH), hypertensive disorders (eclampsia, pre-eclampsia), postpartum infections, obstructed labour and abortion.^3^

One key strategy for reducing pregnancy-related maternal mortality is the provision of compassionate and respectful care during pregnancy.^3,4^ Antenatal care (ANC) provides the opportunity for screening and tests that are directed to identify early and manage pregnancy-related complications that endanger the life and health of the mother and foetus.^4^ It also serves as a foundation for linking pregnant women to other healthcare services when necessary.^2–4^ However, the uptake of adequate ANC (at least four visits) and timely (early in the first trimester) ANC services remains low in SSA. Only 53% of pregnant mothers in SSA received four or more ANC visits in 2020.^5^

A host of factors may deter mothers from accessing adequate and timely ANC. Intimate partner violence (IPV) is one such factor.^6–12^ A meta-analysis of 10 studies in Africa indicated that women who experienced IPV had 25% decreased odds of accessing adequate ANC.^6^ In other studies, women who experienced physical and emotional IPV were 52%, 27% and 32% less likely to receive adequate ANC.^9–11^ Furthermore, IPV predicts late entry into ANC (past the 16th week of pregnancy); women who recently experienced sexual IPV were 1.55 times more likely to enter ANC late.^12^

Research findings are not consistent, however. A study in Nepal found no significant association between IPV and the uptake of skilled maternity care.^13^ In India, no significant association was observed between emotional IPV and reproductive health outcomes.^14^ An Egyptian study indicated that ever-abused professional women were more likely to have four or more ANC visits than those who did not report abuse.^15^ And a study in Rwanda found no significant association between IPV and adequate ANC.^16^

Women who experience IPV are also more likely to have low-birthweight babies, induced abortions, depression and acquire human immunodeficiency virus or syphilis.^7^ They are also more likely to experience unwanted pregnancy, miscarriage and stillbirth.^4,17,18^ More than one-third of the women intentionally killed globally in 2017 were killed by their current or former intimate partner.^19^ A study in South Africa found that of 3797 female homicides, 50.3% were from IPV.^20^ Children who witness IPV are also more likely to develop anxiety, depression, poor school performance, low self-esteem, nightmares, physical health complaints and, in extreme cases, death.^21–23^

IPV is defined as any behaviour within an intimate relationship that causes physical, psychological/emotional or sexual harm to those in the relationship.^7,8^ It is the most prevalent form of violence against women worldwide.^7,8^ Nearly 37% of ever-partnered women in Africa experienced some form of physical and/or sexual violence from their intimate partner, which is slightly higher than the 30% estimate globally.^7,8^

Many theoretical perspectives and models have been developed to explain and predict IPV risk. From a feminist model perspective, patriarchy and male dominance are seen as primary drivers.^24–26^ From a sociological model perspective, one's prior experiences of violence and unequal resources in relationships are viewed as primary predisposing factors for violence.^24–26^ In contrast, the social ecologic model provides a more comprehensive and nuanced perspective by mapping IPV drivers to individual, relationship, community and societal levels of influence.^27,28^

Ethiopia has significantly reduced maternal mortality over the last decade.^1,2^ However, it is still ranked among the highest burdened countries globally.^29^ In 2017, 14 000 Ethiopian mothers died.^1,2^ The country also has a high maternal mortality ratio (MMR) of 401 deaths per 100 000 live births. This is nearly double the global MMR of 211.^1,2^ Annually, almost 11% of Ethiopian mothers experience adverse pregnancy outcomes.^29^ Despite these high burdens of mortality and morbidity, the 2016 Ethiopia Demographic and Health Survey (EDHS) report indicated only 32% of mothers received adequate ANC nationally.^29^ Further, 37% of Ethiopian mothers did not receive any ANC.^29^ Simultaneously, the report showed IPV remains high, with 27% of reproductive-age women reporting a current experience of IPV and 34% reporting ever having experienced IPV.^29^

### Predicting health service utilization with continuous IPV scores

Usually, measurement of maternal IPV experience uses multiple items mapped to three subscales for emotional, sexual and physical violence. The EDHS ‘domestic violence module’ measures IPV experience using 13 items mapped to these three subscales.^29^

However, violence-related studies to date have primarily analysed IPV as a dichotomous variable—‘yes’ she experienced IPV or ‘no’ she did not. This yes/no dichotomous approach merges and treats a woman victim of only a single form and/or number of violent behaviours equally with the woman who experienced multiple forms and numbers of violent behaviours from emotional, sexual and physical partner violence. Treating IPV as a dichotomous ‘yes’ versus ‘no’ experience may dilute the impact of IPV (overall and the three subscales) on the outcome variable of interest, including its effect on adequate ANC utilization and other health services.

Historic approaches to dichotomizing IPV and not measuring it on multiple scales may explain in part why studies on IPV and obstetric health service utilization have mixed findings. A study done to assess how different conceptualization and coding of IPV variables affects interpretation of the impact of violence prevention intervention compared the ‘standard’ dichotomous approach to a new method that accounted for the severity and intensity of IPV subscales.^30^ The study indicated the new approach allowed for a better understanding of the impacts of the intervention, which was masked by the traditional way of lumping maternal violence experience all together. The research used secondary data from six randomized controlled trials conducted in low- and middle-income countries.^30^

Based on the argument and research findings above, this study was designed to treat each type of IPV as discrete count data and measure the potential dose–response relationships between each IPV subscale and adequate ANC uptake for the last child born in the 5 y before the survey.

## Methods

### Source of data

The data source for this study was the EDHS 2016 survey, which is de-identified, delinked and publicly available.^29^ The survey was conducted from 18 January to 27 June 2016. The target groups included women ages 15–49 y in randomly selected households across Ethiopia. A stratified multistage cluster sampling technique was used to select samples of enumeration areas (EAs) in two stages. In the first stage, a total of 645 EAs (202 in urban areas and 443 in rural areas) were selected with probability proportional to EA size and with independent selection in each sampling stratum. In the second stage, a fixed number of 28 households per cluster were selected with an equal probability systematic selection from the newly created household listing.^29^ Data collection was done with a face-to-face interview utilizing a computer-assisted personal interviewing data collection system. A total of 15 683 women were interviewed and 95% of women invited to interview provided data.^29^

Information on partner violence was obtained from ever-married women on their experience of violence committed by their current and/or former husbands/partners. Specially constructed weights in the EDHS were used to adjust for the selection of only one woman per household and to ensure that the violence module subsample was nationally representative.^29^

The 2016 EDHS and the corresponding dataset from the DHS program database is the 4th and most recent standard DHS available for public use. In 2019, Ethiopia conducted an interim DHS and collected information on key performance monitoring indicators. However, this mini-DHS did not collect data on domestic violence.

### Measurement of variables

#### Outcome measurement

The outcome variable for this study was adequate utilization of ANC for the last birth that occurred in the 5 y before the survey, defined as four or more ANC services (ANC-4) received from a skilled healthcare provider (such as a doctor, nurse or midwife). Although the current World Health Organization (WHO) recommendations are for pregnant women to have at least eight ANC visits,^4^ the EDHS 2016 data were collected when the focused ANC model, which recommended a minimum of four visits, was the standard model of care.^31^

#### Predictor variable

##### Main predicator variable.

The main predictor variable for this study is IPV measured on three subscales. The 2016 Ethiopia DHS employed 13 items to quantify IPV for currently or ever-partnered women. The emotional violence subscale has three items: say or do something to humiliate you (the woman) in front of others, threaten to hurt or harm you or someone close to you and insult you or make you feel bad about yourself. The sexual violence subscale has three items: physically force you to have sexual intercourse even when you did not want to, physically force you to perform any other sexual acts you did not want to and force you with threats or in any other way to perform sexual acts you did not want to. The physical violence subscale has seven items: push you, shake you or throw something at you; slap you; twist your arm or pull your hair; punch you with his fist or with something that could hurt you; kick you, drag you or beat you up; try to choke you or burn you on purpose; and threaten or attack you with a knife, gun or any other weapon. Thus the IPV score ranges from 0 to 3 for emotional violence, 0–3 for sexual violence and 0–7 for physical violence, with the total IPV score ranging from 0 to 13 without weighting or standardizing the subscale scores.

##### Covariates.

Based on the literature review of factors that have been used to predict ANC service utilization globally and in Ethiopia, maternal sociodemographic variables (e.g. age, religion, region, residence, education and household wealth), obstetrics variables (e.g. last child was wanted, bad obstetric history, number of living children and birth order of the last child), women empowerment variables (e.g. participation in household decision-making) and problems related to healthcare access were included as covariates in this study. In the EDHS, a bad obstetric history is defined as ever experiencing abortion, miscarriage or stillbirth.

The woman empowerment indicator, participation in household decision-making, was a composite variable generated by joining three DHS variables on household decision-making into a single yes/no dichotomous variable. The three merged question items are own healthcare, large household purchases and visits to family or relatives. Each question item was first converted to ‘yes’ if the woman participates in the decision-making alone or jointly with her partner and ‘no’ if she does not participate at all. The other predictor, ‘access to healthcare is a big problem’, was created by merging five variables into a single dichotomous yes/no indicator. These variables were getting medical help for self is a big problem, getting permission to go out is a big problem, getting money needed for treatment is a big problem, distance to the health facility is a big problem and not wanting to go alone to the health facility is a big problem.

### Statistical analysis

SPSS version 27 (IBM, Armonk, NY, USA) was used for data analysis. Data management was done before creating the complex sample analysis plan. We first confirmed each variable's adequacy for use in this study with descriptive figures (histograms, box plots) and descriptive statistics (absolute range, interquartile range [IQR]), median and percentage, since data were not normally distributed). After exploring each variable independently, we explored the relationship between each predictor and the outcome using stratified figures (histograms, box plots) and stratified measures of central tendency and variability (median/IQR or percentage). Next, we statistically tested for differences in the predictors stratified by the outcome (ANC 4 visits versus <4 visits). For continuous variables we used the Wilcoxon ranked sum test to compare the rank distributions across these two levels of ANC uptake. For categorical variables with cell counts >6, we used the χ^2^ test of independence since all variables had cell counts >6 in this large-sample EDHS dataset.

We then quantified the direction and strength of association between each predictor and the outcome of ANC-4 using univariate logistic regression (including the odds ratio [OR], 95% confidence interval [CI] and p-value). We also calculated the area under the receiver operating characteristics curve (AUC, C-statistic) for each individual predictor.

To construct the multivariate logistic regression (MLR) model predicting adequate ANC-4, we empirically and iteratively refined how certain variables were modelled. For example, when continuous variables such as age had a high AUC on univariate modelling but did not have an OR that was statistically significant, we tested various cut points for age based on the ANC-4 stratified box plots and empiric ORs.

After using the empiric approaches to find the optimal cut points, we did a full model MLR by adding all predictor variables along with the IPV subscales treated as continuous count data. Although logistic regression does not require the predictor be unbounded and violation of the assumption of linearity in the logits results in type II errors,^32^ we performed a residual analysis creating a scatter plot of the logit residual by predicted probability and found a linear relationship (see Supplementary Figures S1 and S2). We also compared the sensitivity and the AUC and observed nearly the same model performance for the IPV continuous versus IPV dichotomous model approaches. These findings allowed us to treat the IPV subscales as continuous count data in our final complex samples logistic regression model.^32^ We performed a separate analysis on IPV and ANC for the group who gave birth in the 12 months before the survey and found a 10% improvement in the model sensitivity. We assessed for multicollinearity in the MLR using a variance inflation factor (VIF) cut point >10; variables with a VIF >10 would be considered potentially collinear and subject to being removed from the final MLR model.

Following the data management, complex sample analysis plan was created to predict ANC-4 using three IPV subscales (emotional, sexual and physical), sociodemographic, obstetric and empowerment variables. The EDHS 2016 sample weight for domestic violence, sample strata for sampling errors and clusters variable were used to create the complex sample analysis plan based on how the EDHS data were collected.^29,33^

Complex sample frequency, descriptive and crosstab techniques were used to describe the background characteristics of respondents and test for predicator differences in ANC-4 uptake. Univariate and multivariate complex samples logistic regression analysis with the corresponding ORs and 95% CIs were employed to assess the effect size of each predictor variable on the outcome. Taylor series linearization variance estimation was used to produce design-adjusted standard errors. No observation deletion or suppression was done in the analysis.^34^

Some of the EDHS variables included in this study were specific only for woman currently in a union or living with a man. Thus this analysis was limited to the subpopulation of mothers who were in a union or living with a man at the time of the EDHS.

## Results

### Description of respondents by background characteristics and ANC service utilization

A total of 2599 weighted (2788 unweighted) currently married or in-union mothers from the 2016 EDHS were included in this study. Of the sample, <1% did not have data on ANC. The respondents’ ages ranged from 15 to 49 y, with a mean of 29.8 y. The majority were Christians (60.6%), lived in rural areas (88.1%) and had no formal education (64.3%) (Table 1). Sexual violence was common, with 1 in 10 women (10.3%) reporting having ever experienced it. However, nearly one-quarter of all respondents reported experiences of emotional or physical IPV (23.0% and 22.9%, respectively). Maternal experience of multiple forms of IPV ranged from 5.2% for all subtypes to 14.2% for emotional and physical violence (see Supplementary Figure S3).

**Table 1.** Background characteristics of respondents stratified by adequate ANC uptake for the last child

	Adequate ANC uptake (%) (N=2656, unless indicated)		
Predictors	No	Yes	Total (%)
IPV subscales			
Emotional	χ^2^=26.6, p<0.01		
No	67.7	32.3	77.0
Yes	76.7	23.3	23.0
Sexual	χ^2^=22.0, p<0.01		
No	68.8	31.2	89.7
Yes	78.0	22.0	10.3
Physical IPV	NS		
No	68.7	31.3	77.1
Yes	73.2	26.8	22.9
Sociodemographics			
Age (years)	NS		
15–29	66.9	33.1	50.4
30–39	71.5	28.5	39.9
40–49	77.5	22.5	9.7
Religion	χ^2^=27.9, p=0.01		
Christians	67.0	33.0	60.6
Muslim	72.8	27.2	37.2
Others	93.6	6.4	2.2
Region	χ^2^=89.8, p<0.001		
Agrarian	70.7	29.3	91.5
Pastoralist	77.7	22.3	5.8
City dwellers	22.3	77.7	2.7
Residence	χ^2^=154.3, p<0.001		
Rural	73.7	26.3	88.1
Urban	40.7	59.3	11.9
Woman education level	χ^2^=175.5, p<0.001		
No formal education	77.0	23.0	64.3
Primary	62.3	37.7	26.7
Secondary	43.1	56.9	5.8
Higher	34.8	65.2	3.3
Household wealth quintile	χ^2^=185.9, p<0.001		
Poorest	82.9	17.1	20.9
Poorer	76.5	23.5	22.5
Middle	67.1	32.9	22.3
Richer	70.0	30.0	18.7
Richest	45.9	54.1	15.6
Partner education level^a^	χ^2^=151.0, p<0.001		
No formal education	78.5	21.5	48.6
Primary	66.5	33.5	38.0
Secondary	51.8	48.2	8.4
Higher	40.2	59.8	5.0
Obstetric predictors			
Last child wanted	NS		
Yes	68.3	31.7	75.4
No	74.3	25.7	24.6
Bad obstetric history^b^	NS		
No	69.8	30.4	91.0
Yes	69.3	30.7	9.0
Number of living children	χ^2^=55.4, p<0.001		
0–2	61.9	38.1	35.8
3–5	71.7	28.3	42.1
≥6	78.6	21.4	22.2
Birth order of last child	χ^2^=70.7, p<0.001		
1	60.9	39.1	16.9
2–3	65.0	35.0	30.2
4–5	69.4	30.6	24.0
≥6	80.3	19.7	28.9
Women empowerment predictors			
Participation in household decisions^c^	χ^2^=43.8, p<0.001		
Yes	68.0	32.0	90.0
No	87.0	13.0	10.0
Healthcare access is a big problem	χ^2^=41.3, p<0.001		
Yes	73.0	27.0	74.4
No	60.3	39.7	25.6

ANC uptake and χ^2^ values are rounded to the nearest one decimal place. The p-value is of the adjusted F-statistics. NS: not significant.

^a^N=2641.

^b^Ever experiencing abortion, miscarriage or stillbirth.

^c^N=2615.

Only 30.2% of respondents had utilized adequate ANC services (ANC-4) for their last child. As maternal age and birth order of the last child increased, adequate ANC utilization decreased. In contrast, education level showed a direct positive relationship with adequate ANC utilization for the last child. Maternal age, whether the last child was wanted and maternal experience of a bad obstetric history were found to have no significant relationship with adequate ANC utilization in the χ^2^ test (Table 1).

### Complex sample univariate logistic regression analysis

In the complex samples univariate logistic regression, all the IPV subscales decreased adequate ANC utilization. Physical IPV, however, had a low effect size and was not statistically significant (Table 2).

**Table 2.** Complex sample univariate logistic regression predicting adequate ANC utilization using IPV subscales and covariates

	Complex sample univariate regression result	
Predictors	Crude OR	95% CI
IPV subscales		
Emotional (0–3)	0.74*	0.63 to 0.88
Sexual (0–3)	0.65*	0.50 to 0.80
Physical (0–7)	0.94	0.84 to 1.05
Sociodemographic predictors		
Age category (15–29, 30–39, 40–49 y)	0.78*	0.64 to 0.95
Religion		
Christian	1	
Muslim	0.76	0.54 to 1.07
Others	0.14*	0.04 to 0.48
Region		
Agrarian	1	
Pastoralist	0.69*	0.52 to 0.92
City dwellers	8.40*	5.61 to 12.56
Residence		
Rural	1	
Urban	4.08*	2.87 to 5.80
Woman education, category (none, primary, secondary, higher)	1.97*	1.68 to 2.32
Household wealth quintile, category (poorest, poorer, middle, richer, richest)	1.46*	1.33 to 1.60
Partners education level, category (none, primary, secondary, higher)	1.80*	1.54 to 2.09
Obstetric predictors		
The last child was wanted		
Yes	1	
No	0.74	0.55 to 1.01
Bad obstetric history^a^		
No	1	
Yes	1.02	0.71 to 1.47
Number of living children category (0–2, 3–5, ≥6)	0.66*	0.56 to 0.78
Birth order of last child category (1, 2–3, 4–5, ≥6)	0.73*	0.66 to 0.81
Woman empowerment predicators		
Participation in household decisions		
Yes	1	
No	0.32*	0.19 to 0.52
Healthcare access is a big problem		
Yes	1	
No	1.78*	1.38 to 2.29

The numbers are rounded to the nearest two decimal places.

^a^Ever experiencing an abortion, miscarriage or stillbirth.

*Statistically significant at p<0.05.

### Complex sample multivariate logistic regression predicting ANC-4 utilization

Complex sample multivariate logistic regression was done to measure the effect size of the main predictor variables (three IPV subscales) and related predictors (sociodemographic, obstetric and empowerment) on the uptake of ANC-4 for the last-born child.

Initially, each IPV subscale (controlled for confounders) was tested for its effect on adequate ANC utilization for the last child. Then a full-model complex samples analysis was done by adding all the IPV subscales and covariates into one multivariate model (Table 3). No variables were excluded from the multivariate model due to multicollinearity since all VIFs were <5.

**Table 3.** Complex samples multivariate logistic regression model predicting adequate ANC utilization using IPV subscales and covariates

	Complex sample multivariate logistic regression, full model	
Predictors	aOR	95% CI
IPV subscales		
Emotional (0-3)	0.78*	0.64 to 0.97
Sexual (0-3)	0.68*	0.50 to 0.92
Physical (0-7)	1.15	0.97 to 1.35
Sociodemographics		
Age (15–29, 30–39, 40–49 y)	1.04	0.77 to 1.41
Religion		
Christians	1	
Muslim	1.03	0.71 to 1.48
Other	0.22*	0.06 to 0.78
Region		
Agrarian	1	
Pastoralist	0.76	0.52 to 1.11
City dwellers	2.81*	1.63 to 4.86
Residence		
Rural	1	
Urban	1.37	0.77 to 2.43
Woman education level (none, primary, secondary, higher)	1.22	0.95 to 1.55
Household wealth quintiles (poorest, poorer, middle, richer, richest)	1.17*	1.03 to 1.32
Partners education level (none, primary, secondary, higher)	1.26*	1.02 to 1.56
Obstetric predictors		
The last child was wanted		
Yes	1	
No	0.81	0.58 to 1.15
Bad obstetric history^a^		
No	1	
Yes	1.18	0.78 to 1.78
Number of living children (0–2, 3–5, ≥6)	1.00	0.70 to 1.45
Birth order of the child (1, 2–3, 4-5, ≥6)	0.90	0.70 to 1.15
Women empowerment predictors		
Participation in household decisions		
Yes	1	
No	0.44*	0.26 to 0.73
Healthcare access is a big problem		
Yes	1	
No	1.07	0.79 to 1.45

The numbers are rounded to the nearest two decimal places.

^a^Ever experiencing an abortion, miscarriage or stillbirth.

*Statistically significant at p<0.05.

The result from the full-model regression indicated a dose–response relationship between IPV subscale scores and ANC-4. A unit increase in the emotional IPV score from 0 through 3 decreased the likelihood of ANC-4 by 22.0% (aOR 0.78 [CI 0.64 to 0.97]) and a unit increase in the sexual IPV score from 0 to 3 decreased ANC-4 by 32.0% (aOR 0.68 [CI 0.50 to 0.92]). On the other hand, a unit increase in the physical IPV score from 0 to 7 increased ANC uptake slightly, although the change was not significant (aOR 1.15 [CI 0.97 to 1.35]) (Table 3).

## Discussion

This study measured the impact of three forms of maternal IPV on the uptake of adequate ANC. We modelled each IPV subscale (sexual, emotional and physical) as continuous count data to quantify for any dose–response relationship between the three forms of IPV and ANC-4 using complex samples logistic regression. To our knowledge, no previous studies have measured the dose–response relationship between each type of IPV and ANC-4.

We identified a significant inverse dose–response relationship between both emotional and sexual IPV and adequate ANC service utilization for the last child born in the 5 y before the survey. Our finding that emotional violence deters ANC-4 utilization is not consistent with other studies,^9,14,16^ nor is the finding for sexual violence.^10,16^ These studies indicated no significant association between IPV subscales and maternal health service utilization. However, the studies followed the usual way of dichotomizing IPV as a whole or for each subscale, as opposed to our continuous count data approach. Research indicates that lumping together all maternal violence experiences could potentially hamper the expression of subtle predictor–outcome relationships.^30^ We believe that variation in conceptualization and coding of IPV partially explains the discrepancy of the research findings and could be attributed to differences in sample size, type of study population and sociocultural factors.

Our research discovery of a predictive dose–response relationship between IPV subscales and ANC uptake provides a novel approach in quantifying IPV and demonstrating how both sexual and emotional IPV decrease ANC utilization. Further, this study uniquely quantifies the differential impact of each form of IPV on ANC-4. The approach allows researchers to more clearly measure the relationship between various forms of IPV and health service utilization. Using ordinal IPV subscales advances existing science on IPV and health service utilization. Historically, researchers often lumped together maternal experience of violence into a single dichotomous yes/no variable.^9–16^

The physical, psychological, social and other negative impacts of IPV are not limited to the woman victim only. Children and other family members who lived through and experienced such a violent relationship may be affected by its direct and/or indirect consequences.^21–23^ The finding in this study that IPV has a dose–response relationship with maternal ANC service utilization suggests, by extension, that other IPV-related health outcomes may also have a similar dose–response relationship with different forms of IPV.

Although not significant, a unit increase in physical IPV experience was found to increase the uptake of ANC-4. In contrast, emotional and sexual IPV decreased ANC-4. One possible explanation for such a contradictory outcome could be that physical harm from IPV may motivate mothers to seek medical help for themselves. Another possible explanation for such a relationship may be the moderation effect of other covariates. If this is true, the effect of physical IPV on ANC utilization may be moderated by a third variable or complex set of other variables. Cultural and social influences may also play a role in the differential impact of various forms of IPV on ANC-4 uptake.

There are different theories and models that explain how IPV experience predisposes the victim to a variety of untoward health conditions. Among others, the abuser's psychological control of the woman where the perpetrator makes the victim financially dependent or limits the ability to control her sexual and reproductive decision-making is cited as one reason.^7^ In this study, the combination of a high prevalence of IPV coupled with low utilization of adequate ANC suggests women in Ethiopia are experiencing multifaceted abuse. This warrants intensified work to increase empowerment, gender equity and creating community-level IPV awareness that could support ANC-4 uptake.

Existing literature to date defines IPV as ‘severe’ if the victim experienced certain aspects of physical violence (i.e. having been kicked or dragged, strangled or burned or threatened with a knife/gun or other weapon), although some authors consider any sexual violence as severe.^7,8^ However, the novel dose–response measurement of IPV using three subscales could expand the definition of severe IPV to account for both the number and type of IPV behaviours experienced. Future studies could test various combinations of IPV experiences and/or various dose cut points for defining ‘severe’ IPV. Additional studies could create short-forms for rapidly screening pregnant women for each form of IPV in routine clinical practice.

We found a 10% improvement in sensitivity when we modelled IPV and adequate ANC utilization for women who gave birth in the 12 months before the survey. Apart from the difference in sample size (2599 vs 917) and magnitude of ANC-4 utilization (30.2% vs 34.3%), this may in part be that for some women who gave birth in the 5 y before the survey, IPV might have occurred after delivery of the last child.

### Strengths and limitations of the study

This study used nationally representative data from the EDHS 2016. Thus the findings from this research can be generalized to Ethiopia. The study assesses the dose–response relationship of IPV with ANC-4 utilization, making it the first known study of the dose–response impact of IPV on a health outcome. Yet, since EDHS data are retrospective and cross-sectional, it is not possible to establish a definitive cause–effect relationship between IPV and ANC-4 uptake.

## Conclusions

The findings of this study showed that as the number of maternal experiences of emotional and sexual IPV increases, the probability of accessing adequate ANC services (ANC-4) decreases. Consequently, the number and types of IPV (emotional, sexual and physical) should be assessed and responded to differently when used to predict adequate ANC.

Based on our finding of a dose–response relationship between the IPV subscale and ANC-4, we believe that each woman victim of IPV should be managed individually. We therefore suggest that IPV exposure dose and exposure type require assessment in order to mobilize differential interventions in the prevention, management and control of IPV.

Additional studies to assess for the presence of a dose–response relationship between IPV subscales and other maternal–child health services and outcomes are needed. Further research work to create ‘severe’ IPV indicators and short-forms for screening for IPV in routine clinical practice are warranted.

## Supplementary Material

ihaf003_Supplemental_Files

## Data Availability

The dataset used for this study is available in the DHS repository (https://dhsprogram.com) upon registration and submission of research protocols. For further information about the data, please visit: https://dhsprogram.com/data/Access-Instructions.cfm.
