# Supplementary material for: Intimate partner violence and maternal antenatal care utilization: is there a dose-response relationship? Findings from the Ethiopian National Demographic and Health Survey
Source: Int Health. 2025 Feb 5;17(4):542–51. doi: 10.1093/inthealth/ihaf003 (PMC12212190; doi:10.1093/inthealth/ihaf003)
Supplement: ihaf003_Supplemental_Files [file ihaf003_supplemental_files.zip › Supplementary Figure 3.docx]

**Fig 3**: Venn diagram showing prevalence of ever experience of IPV.

**S & P: 6.6**

**All: 5.2**

**Em & P:14.2**

**Em & S: 6.3**

**Key:** Em: Emotional IPV, S: Sexual IPV, P: Physical IPV, All: all types of IPV subscale
