# Supplementary figures and images for: Intimate partner violence and maternal antenatal care utilization: is there a dose-response relationship? Findings from the Ethiopian National Demographic and Health Survey
Source: Int Health. 2025 Feb 5;17(4):542–51. doi: 10.1093/inthealth/ihaf003 (PMC12212190; doi:10.1093/inthealth/ihaf003)

Scatter plot of residuals by Predicted probability, and Venn diagram


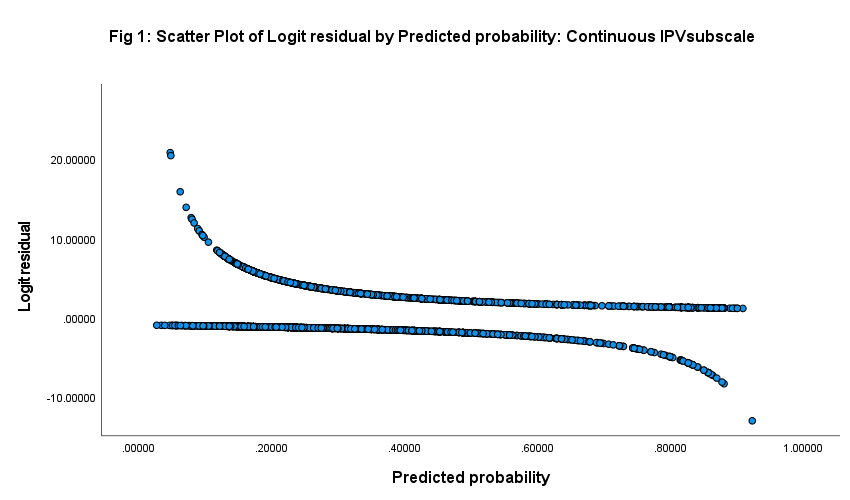


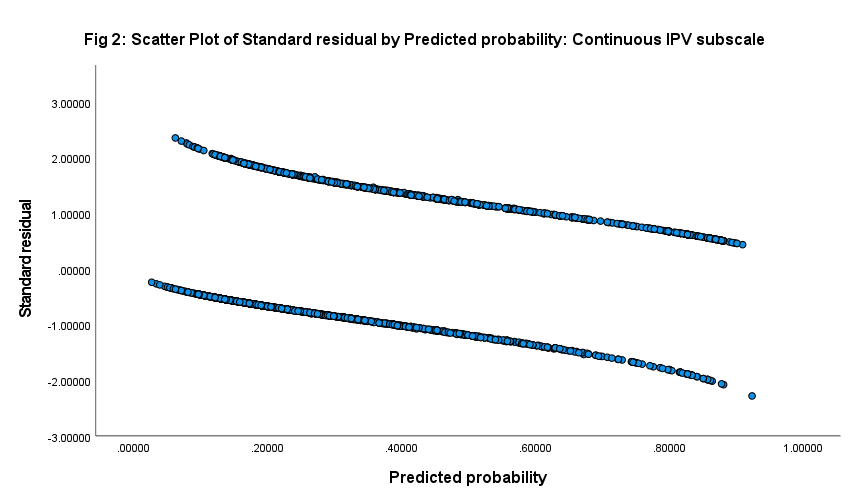

Supplement: ihaf003_Supplemental_Files [file ihaf003_supplemental_files.zip › Supplementary Figure 1 and 2.docx]
